# Disease Burden of Chronic Kidney Disease Due to Hypertension From 1990 to 2019: A Global Analysis

**DOI:** 10.3389/fmed.2021.690487

**Published:** 2021-06-21

**Authors:** Aiming Chen, Minjie Zou, Charlotte Aimee Young, Weiping Zhu, Herng-Chia Chiu, Guangming Jin, Lin Tian

**Affiliations:** ^1^Department of Pharmacy, Fifth Affiliated Hospital, Sun Yat-sen University, Zhuhai, China; ^2^State Key Laboratory of Ophthalmology, Zhongshan Ophthalmic Center, Sun Yat-sen University, Guangzhou, China; ^3^Department of Ophthalmology, Third Affiliated Hospital, Nanchang University, Nanchang, China; ^4^Department of Nephrology, Fifth Affiliated Hospital, Sun Yat-sen University, Zhuhai, China; ^5^Institute for Hospital Management, Tsinghua University, Beijing, China; ^6^Department of Healthcare Administration and Medical Informatics, Kaohsiung Medical University, Kaohsiung, Taiwan; ^7^Department of Health Policy and Management, Bloomberg School of Public Health, John Hopkins University, Baltimore, MD, United States

**Keywords:** chronic kidney disease, hypertension, global disease burden, time trends, associated factors

## Abstract

**Background:** Although it is widely known that hypertension is an important cause of chronic kidney disease (CKD), little detailed quantitative research exists on the burden of CKD due to hypertension.

**Objective:** The objective of the study is to estimate the global disease burden of CKD due to hypertension and to evaluate the association between the socioeconomic factors and country-level disease burden of CKD due to hypertension.

**Methods:** We extracted the disability-adjusted life-year (DALY) numbers, rates, and age-standardized rates of CKD due to hypertension from the Global Burden of Disease Study 2019 database to investigate the time trends of the burden of CKD due to hypertension from 1990 to 2019. Stepwise multiple linear regression analysis was performed to evaluate the correlations between the age-standardized DALY rate and socioeconomic factors and other related factors obtained from open databases.

**Results:** Globally, from 1990 to 2019, DALY numbers caused by CKD due to hypertension increased by 125.2% [95% confidential interval (CI), 124.6 to 125.7%]. The DALY rate increased by 55.7% (55.3 to 56.0%) to 128.8 (110.9 to 149.2) per 100,000 population, while the age-standardized DALYs per 100,000 population increased by 10.9% (10.3 to 11.5%). In general, males and elderly people tended to have a higher disease burden. The distribution disparity in the burden of CKD due to hypertension varies greatly among countries. In the stepwise multiple linear regression model, inequality-adjusted human development index (IHDI) [β = −161.1 (95% CI −238.1 to −84.2), *P* < 0.001] and number of physicians per 10,000 people [β = −2.91 (95% CI −4.02 to −1.80), *P* < 0.001] were significantly negatively correlated with age-standardized DALY rate when adjusted for IHDI, health access and quality (HAQ), number of physicians per 10,000 people, and population with at least some secondary education.

**Conclusion:** Improving the average achievements and equality of distribution in health, education, and income, as well as increasing the number of physicians per 10,000 people could help to reduce the burden of CKD due to hypertension. These findings may provide relevant information toward efforts to optimize health policies aimed at reducing the burden of CKD due to hypertension.

## Introduction

Chronic kidney disease (CKD) is defined as an abnormality in the kidney structure or function present for >3 months (1), and has been a public health concern worldwide in recent years (1–3). From 1990 to 2017, the global all-age prevalence of CKD increased by 29.3% [95% uncertainty interval (UI), 26.4 to 32·6%] to 697.5 million (95% UI, 649.2 million to 752.0 million), and all-age mortality increased by 41.5% (95% UI, 35·2 to 46·5%) to 1.2 million (95% UI, 1.2 to 1.3 million) (4). CKD has risen from the 29th leading cause of global disability-adjusted life-years (DALYs) in 1990 for all ages to the 18th in 2019 (5). Moreover, the early stages of CKD are usually asymptomatic, and patients' awareness of CKD tend to be low (6–11), which makes the prevention, early detection, and early treatment of this disease particularly important.

Hypertension has been identified as a major driver of CKD (4, 12–15), and from 1975 to 2015, the number of adults with hypertension increased by 536 million (16). Particularly, in East Asia, Eastern Europe, tropical Latin America, and western Sub-Saharan Africa, hypertension accounted for the largest proportion of the disease burden of CKD (4). Although the burden of hypertension has increased significantly, the global importance of CKD due to hypertension has not been widely recognized, and there have been few quantitative studies on the burden of CKD due to hypertension in the past decades.

To estimate the magnitude of the disease burden of CKD due to hypertension and to provide information for disease prevention and control, we utilized DALYs, a public health metric widely used to evaluate and compare the overall disease burden of different diseases (4, 5, 17–19). DALYs were obtained from the Global Burden of Disease, Injuries, and Risk Factors Study (GBD) 2019 to investigate the time trends and associated socioeconomic factors of CKD due to hypertension.

## Materials and Methods

### Measures for the Global Disease Burden of Chronic Kidney Disease Due to Hypertension

Data on DALYs of CKD due to hypertension were extracted from the GBD 2019 on the Global Health Data Exchange (GHDx) (http://ghdx.healthdata.org/gbd-results-tool, accessed 30 Oct 2020), which provides data on global, regional, and national DALYs for 369 diseases and injuries, and 87 risk factors in 204 countries and territories from 1990 to 2019. A detailed research methodology of GBD 2019 has been published in previous studies (5, 15, 19, 20). We calculated DALYs as the sum of years lived with disability (YLD) and the years of life lost (YLL) due to premature death caused by CKD due to hypertension expressed by the formula: DALYs = (number of prevalent cases × disability weight) + (number of deaths × standard life expectancy at age of death in years) (14, 15, 21). The DALY rate was calculated as the number of cases per 100,000 population, and the age-standardized DALY rate was adjusted according to the population size and age structure, which was reported in the GHDx (https:// ghdx.healthdata.org, accessed 30 Oct 2020). The global map of disease burden distribution was also obtained from GHDx (https://vizhub.healthdata.org/gbd-compare/, accessed 30 Oct 2020).

We extracted the following data from 1990 to 2019 for further analysis: (i) global DALY numbers, rates, age-standardized rates, and age-standardized rates for different World Health Organization (WHO) regions; (ii) global age- and gender-specific DALY numbers; and (iii) DALY numbers, rates, and age-standardized rates in different countries.

### Selection of Country-Level Factors

According to published GBD studies on different chronic diseases (22–24), different country-level demographic and socioeconomic factors were selected from open databases to investigate their correlations with disease burden of CKD due to hypertension. Socio-demographic index (SDI), a composite index reflecting a country's socio-demographic development status, was also extracted from the GHDx (http://ghdx.healthdata.org, accessed 10 Nov 2019). SDI is the geometric mean of 0 to 1 indices of total fertility rate under the age of 25, lag distributed income per capita, and mean education for those aged 15 and older. Countries were divided into five groups by the SDI value: low SDI, middle-low SDI, middle SDI, middle-high SDI, and high SDI. Human development index (HDI) was collected from the United Nations Development Programme (UNDP) database (http://hdr.undp.org/en/data, accessed 10 Nov 2019), which is a summary measurement of average achievements in three basic dimensions of human development based on four indicators: leading a long and healthy life (life expectancy at birth), being knowledgeable (expected years of schooling and mean years of schooling), and enjoying a decent standard of living (Gross National Income per capita). On a scale of 0 (worst) to 1 (best), countries were classified into four categories by their value: low (<0.550), medium (0.550–0.699), high (0.700–0.799), and very high (0.800 or greater) HDI. In addition, to further comprehend these distributions, inequality-adjusted human development index (IHDI) collected from UNDP (http://hdr.undp.org/en/data, accessed 10 Nov 2019) was also included as a variable. Additionally, the proportion of people receiving at least some secondary education (aged 25 and older) is included as a national educational level index (http://hdr.undp.org/en/data, accessed 10 Nov 2019). The health access and quality (HAQ) index extracted from the GHDx (http://ghdx.healthdata.org, accessed 10 Nov 2019) is a single, interpretable novel measure, which developed to improve and expand the comparative assessment of the availability and quality of personal health care services across the development process (25). The number of physicians per 10,000 people, reflecting a country's healthcare infrastructure was also obtained from UNDP (http://hdr.undp.org/en/data, accessed 10 Nov 2019) and selected as a potential associated factor with disease burden of CKD due to hypertension.

### Statistical Analysis

The difference in DALY numbers between sexes in 1990 and 2019 was compared by the Wilcoxon signed-rank test. Kruskal–Wallis H-test was conducted to assess the difference in age-standardized DALY rates across HDI-based categories, followed by Mann–Whitney U-test for *post-hoc* pairwise comparisons. Stepwise multiple linear regression analysis was conducted to explore the associations between the age-standardized DALY rates of CKD due to hypertension and country-level demographic and socioeconomic factors; commonly used country-level indexes were selected as potential associated factors with CKD attributable to hypertension based on previous published studies (23, 24, 26) and variables at a level of *p* ≤ 0.2 were selected for inclusion in multiple stepwise regression analysis for further analysis. All statistical analyses were performed by using Stata MP 15.0 (Stata Corp LP, College Station, Texas, USA). Figures were drawn by GraphPad Prism software (version 5.01, GraphPad Software; San Diego, CA, USA). A *p*-value < 0.05 was considered to be statistically significant unless otherwise specified.

## Results

### Trends in the Global Disease Burden of Chronic Kidney Disease Due to Hypertension

**Figure 1:**
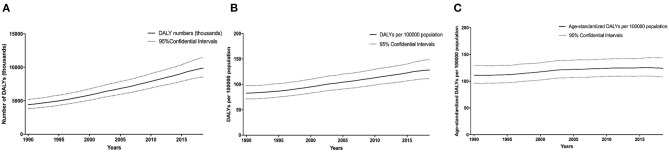
Time trends in the global disease burden of CKD due to hypertension in 1990–2019. DALY numbers **(A)**, DALY rates **(B)**, and age-standardized DALY rates **(C)**. Dashed lines represent 95% confidence intervals. CKD, chronic kidney disease; DALY, disability-adjusted life-year.

Globally, the number of DALYs caused by CKD due to hypertension increased by 125.2% [95% confidential interval (CI), 124.6 to 125.7%], rising from 4,424.4 (3,817.9 to 5,211.1) in 1990 to 9,962.4 (8,582.3 to 11,544.1) in 2019 (Figure 1A). During the same period, the DALY rate increased by 55.7% (55.3 to 56.0%) to 128.8 (110.9 to 149.2) per 100,000 population (Figure 1B), while the age-standardized DALYs per 100,000 population increased by 10.9% (10.3 to 11.5%) (Figure 1C).

### Global Disease Burden of Chronic Kidney Disease Due to Hypertension by Age and Gender

**Figure 2:**
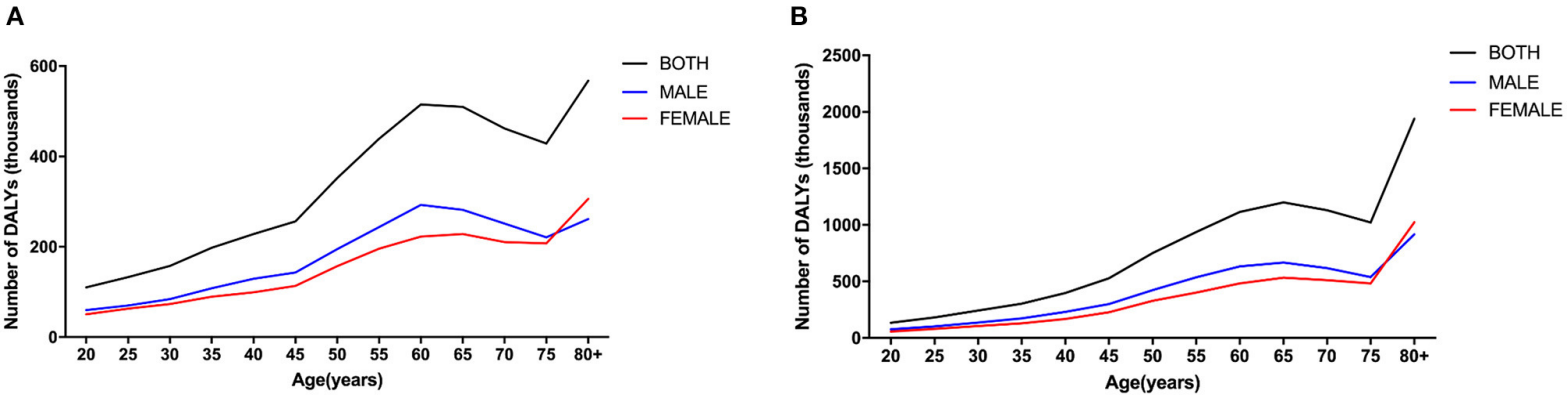
Age- and gender-specific global disease burden of CKD due to hypertension. DALY numbers in 1990 **(A)**, and DALY numbers in 2019 **(B)**. CKD, chronic kidney disease; DALY, disability-adjusted life-year.

In 1990, the number of DALYs for males surpassed those for females before approximately 75 years old and then was reversed. In 2019, the trend for males and females was similar to that of 1990. In 1990 and 2019, the trend in the number of DALYs caused by CKD due to hypertension with age was similar in both sexes in total (Figure 2).

### Distribution Disparity in the Burden of Chronic Kidney Disease Due to Hypertension

**Figure 3:**
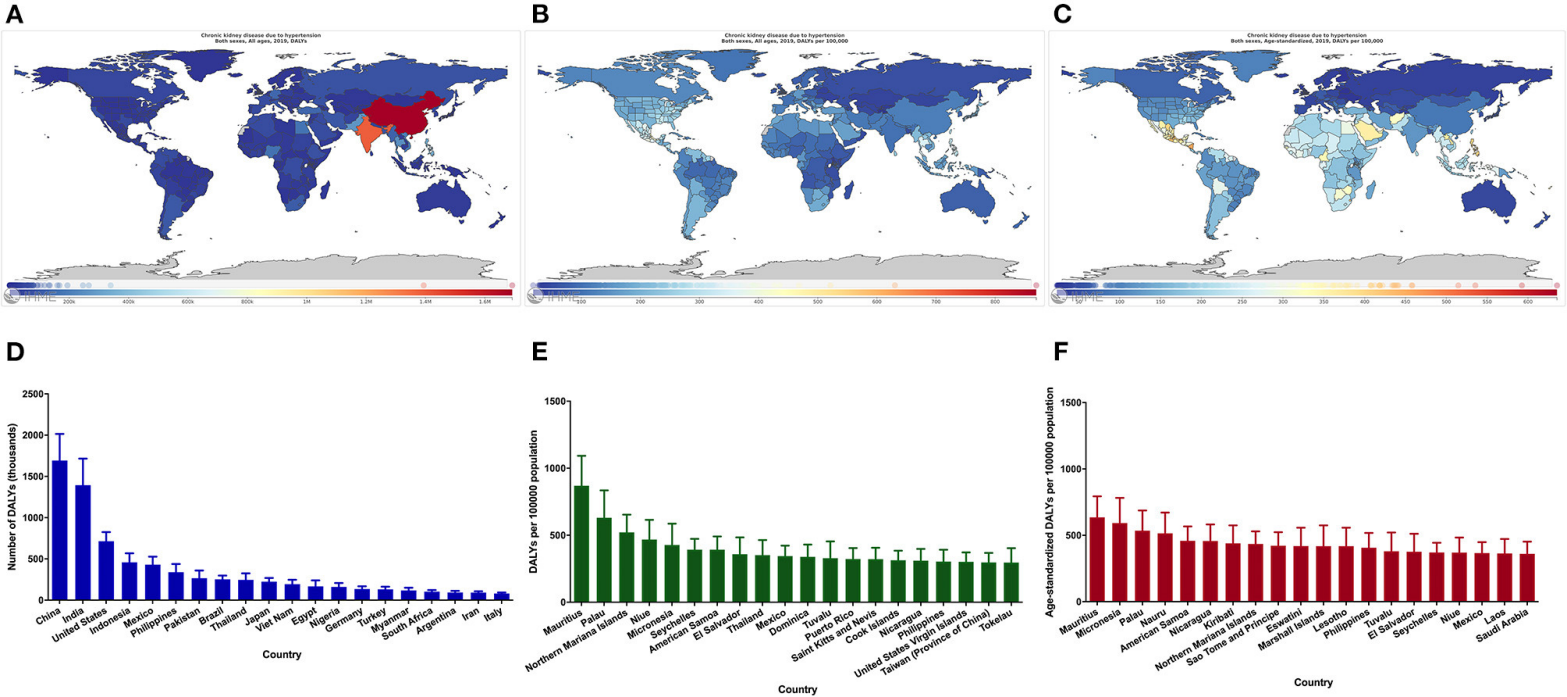
Global map of the disease burden of CKD due to hypertension and the 20 countries with the highest burden. DALY numbers **(A)**, DALY rates **(B)**, Age-standardized DALY rates **(C)**, The 20 countries with the highest DALY numbers **(D)**, The 20 countries with the highest DALY rates **(E)**, The 20 countries with the highest age-standardized DALY rates **(F)**. CKD, chronic kidney disease; DALY, disability-adjusted life-year.

Considerable disparity was illustrated in the burden of CKD due to hypertension, with DALY numbers varying more than 16-fold between countries (Figure 3A). India and China had the highest DALY numbers in 2019, with more than 1.3 million DALY numbers (Figures 3A,D). After adjusting for population size, Mauritius had the highest rate of DALYs, with DALYs for CKD due to hypertension of more than 860 per 100,000 population (Figures 3B,E). After adjusting for population size and age, Mauritius still had the highest burden, with more than 630 DALYs per 100,000 population (Figures 3C,F).

### Burden by World Health Organization Regions, Income, and Socio-Demographic Index

**Figure 4:**
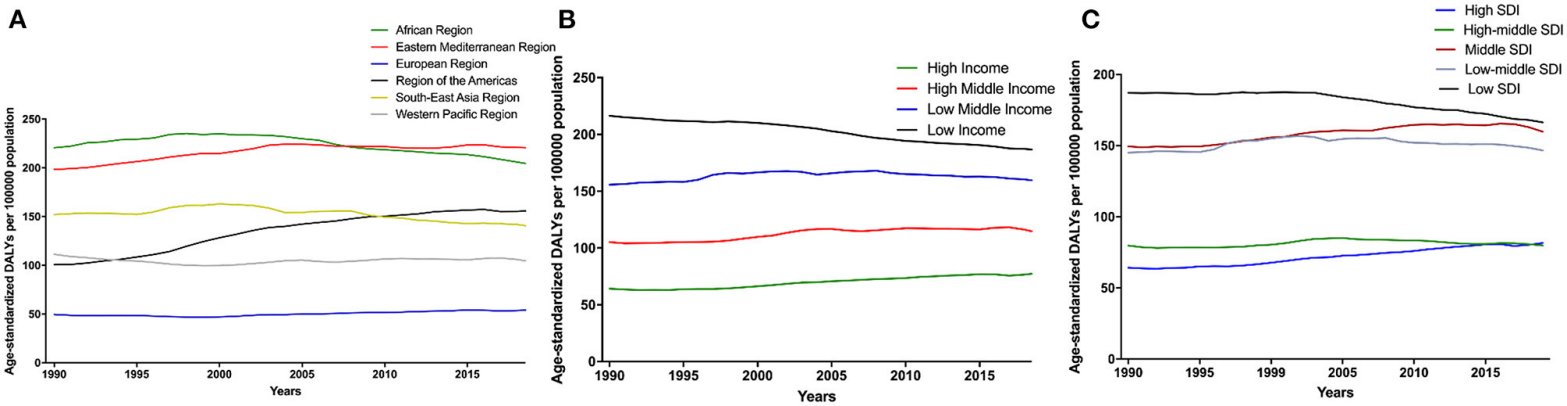
Time trends of global disease burden of CKD due to hypertension in 1990–2019 among different regions **(A)**, Global disease burden of CKD due to hypertension by different levels of income regions **(B)**, and age-standardized DALY rate by different levels of SDI regions **(C)**. CKD, chronic kidney disease; DALY, disability-adjusted life-year. SDI, socio-demographic index.

The age-standardized DALY rate of CKD due to hypertension was high in the African region and Eastern Mediterranean region, and the lowest and most stable in the European region between 1990 and 2019 (Figure 4A). In terms of income levels of comparing the burden of disease, regions with lower incomes tended to have higher burden of disease (Figure 4B). Analysis of the age-standardized DALY rate according to the SDI classification showed that the burden of CKD due to hypertension was more significant in low, low-middle, and middle SDI regions, and relatively lower in high and high-middle SDI regions (Figure 4C).

### Socioeconomic Factors Associated With Country-Level Disease Burden of Chronic Kidney Disease Due to Hypertension

As shown in Table 1, in univariate linear regression, SDI, HDI, IHDI, HAQ, physicians per 10,000 people, and population with at least some secondary education had significant negative association with the age-standardized DALY rates of CKD due to hypertension. The number of physicians accounted for 36.9% of global variations in age-standardized DALY rates of CKD due to hypertension. For stepwise multiple regression analysis in model 1, which adjusted for SDI, HAQ, number of physicians, and population with at least some secondary education, HAQ and number of physicians per 10,000 people were associated with the age-standardized DALY rates of CKD due to hypertension. In model 2, which adjusted for HAQ, number of physicians per 10,000 people, and four components of HDI, three indicators (HAQ, number of physicians per 10,000 people, and life expectancy at birth) were associated with age-standardized DALY rates of CKD due to hypertension. IHDI and number of physicians per 10,000 people were associated factors with age-standardized DALY rates of CKD due to hypertension significant in Model 3 (IHDI, HAQ, number of physicians, and population with at least some secondary education were adjusted).

**Table 1:** Linear regression analysis of the relationship between the country-level disease burden of CKD due to hypertension and the socioeconomic variables.

	**R square**	***P*-value**	**β (95%CI)**
**Univariate analysis**			
SDI	0.225	<0.001	−314.5 (−399.0 to −230.1)
HDI	0.200	<0.001	−347.9 (−450.0 to −245.8)
IHDI	0.332	<0.001	−273.4 (−334.9 to −211.9)
HAQ	0.304	<0.001	−2.92 (−3.55 to −2.28)
Physicians (per 10,000 people)	0.369	<0.001	−4.72 (−5.66 to −3.78)
Population with at least some secondary education	0.271	<0.001	−2.08 (−2.63 to −1.52)
**Multivariate analysis [only variates with significant association**			
**(*****p*** **<** **0.05) are shown]**			
Model 1^*^	0.420	<0.001	
HAQ			−3.06 (−5.47 to −0.64)
Physicians (per 10,000 people)			−2.51 (4.15, −0.86)
Model 2^**^	0.433	<0.001	
HAQ			−3.99 (−6.07 to −1.90)
Physicians (per 10,000 people)			−2.60 (−4.20 to −0.99)
Life Expectancy at Birth			5.88 (1.57 to 10.18)
Model 3^***^	0.461	<0.001	
IHDI			−161.1 (−238.1 to −84.2)
Physicians (per 10,000 people)			−2.91 (−4.02 to −1.80)

*SDI, socio-demographic index; HDI, human development index; IHDI, inequality-adjusted HDI; HAQ, health access and quality Index*.

* *Model 1: adjusted for SDI, HAQ, number of physicians, and population with at least some secondary education*.

** *Model 2: adjusted for HAQ, number of physicians, and components of HDI (mean years of schooling, expected years of schooling, gross national income per capita, and life expectancy at birth)*.

*** *Model 3: adjusted for IHDI, HAQ, number of physicians, and population with at least some secondary education*.

## Discussion

Globally, DALY numbers and DALY rate of CKD due to hypertension showed a rising trend from 1990 to 2019, which reached 9,962.4 (8,582.3 to 11,544.1) and 128.8 (110.9 to 149.2) per 100,000 population in 2019, respectively, while the age-standardized DALYs per 100,000 population stayed relatively steady. In addition, this study suggests there were significant substantial disparities in sexes, countries, WHO regions, income, and SDI, and socioeconomic factors for disease burden caused by CKD due to hypertension.

Presently, there is insufficient public awareness of CKD worldwide (6–11). A study in the United States found that awareness of CKD, including in high-risk groups, has been low—even lower than that of hypertension and diabetes during the same period (27). In a cross-sectional study that compared CKD with other common chronic diseases, physicians discussed CKD with patients infrequently and tended to use medical jargon, which have decreased the patient's understanding and awareness of CKD. In a primary care setting, increasing the frequency and emphasis of CKD in physician–patient discussions can improve the clinical outcomes of patients (28). Additionally, challenges perceived by primary care providers, such as their lack of adequate knowledge or skills, fear of overwhelming patients, time limits on patient visits, lack of reimbursement for CKD patient education, and lack of educational resources, were illustrated in another study (29). To elevate kidney disease awareness, the National Kidney Foundation, American Society of Nephrology, and US Department of Health and Human Services launched the Public Awareness Initiative for Advancing American Kidney Health (National Kidney Foundation. The public awareness initiative for AAKH was announced as a public-private partnership with NKF and ASN. November 4, 2019. https://www.kidney.org/news/aakhannounced-public-private-partnership-nkf-asn. Accessed October 10, 2020). In 2019, the State Council of the People's Republic of China issued their statement on the implementation of health action in China (http://www.gov.cn/zhengce/content/2019-07/15/content_5409492.htm). This action emphasized the promotion of health literacy as the premise of improving the health of its people, formally referred to as the “health knowledge popularization action.” This action also combines health popularization with a focus on performance appraisal of medical institutions and relevant management departments. These measures were aimed to encourage individuals, medical staff, as well as society and the government at large to be proactive in increasing the awareness of CKD due to hypertension. Overall, the current global burden of CKD indicates a need for a multi-faceted public health initiative to improve the current standards for screening, prevention, and treatment.

Our results show that males have a greater burden of disease than females before the age of 75 (both in 1990 and 2019). This finding may be due to a higher prevalence of hypertension in adult males than in females of the same age group before the age of 75 but lower in adult males than in females after that time point, as shown in a previous study (30). In addition, the overall hypertension control rate of women is higher than that of men as reported by previous studies (30–32). Our results also show that the disease burden of CKD due to hypertension in 2019 was substantially higher than that in 1990, which may be related to the increased prevalence of hypertension (16, 33).

We found that the burden of CKD due to hypertension was skewed more toward low-income and low-SDI countries. This phenomenon may be attributed to high prevalence and low awareness of chronic kidney disease, and a significant proportion of people who were aware of their disease but did not receive treatment in these countries, a result that suggests that increased effort should be made in CKD prevention and treatment in these countries and regions (11). A study also revealed an inverse relationship between age-standardized disability adjusted annual rate of life and measures of health care access and quality, which suggests the necessity of identifying related factors (34) for reducing the burden of CKD due to hypertension and developing a comprehensive action plan to benefit those at risk or affected by CKD in countries at all levels of the development spectrum (35).

The result of the stepwise multiple regression analysis indicated that HAQ was one of the associated factors of disease burden caused by CKD due to hypertension, as countries with high HAQ had relatively low age-standardized DALY rate, while countries with low HAQ had higher age-standardized DALY rate. As in a previous study, this suggests that the burden of CKD is disproportionately born by the countries with the least capacity to respond (14). The number of physicians was also negatively associated with the burden of disease, as increased number of physicians can serve more patients to improve the health of the patient population (29). When the four components of HDI were included in the regression model, life expectancy at birth showed a positive correlation with disease burden of CKD due to hypertension. As age increases, patients with CKD generally have more than one diagnosis of kidney disease and are more likely to develop to late stage CKD. Moreover, the complications, risks, and mortality rate is higher as age increases (36). In a population-based study from Australia, the absolute rate of coronary artery death or nonfatal myocardial infarction was higher in patients with CKD that were 50 years or older than those under 50 years (37). A systematic analysis of changes in CKD burden in the United States from 2002 to 2016 found that 32.3% of the increase in CKD DALY was due to aging (38).

Our results showed that IHDI was significantly negatively correlated with the disease burden of CKD due to hypertension, which indicated that the less inequality present in the three key dimensions of human development such as health, education, and income, the more effective a country can reduce the disease burden of CKD due to hypertension through development. The results suggest that in some developing countries, especially in those with limited resources, poor infrastructure, high cost of CKD treatment, limited labor force and lack of effective health policies, a comprehensive strategy should be taken to provide viable solutions for effectively monitoring kidney health and reduce the disease burden of CKD due to hypertension (39).

The global burden of CKD is rising rapidly and is expected to become the fifth most common cause of life loss in the world by 2040 (40). Hypertension is a main risk factor leading to CKD; therefore, taking measures to reduce the burden of CKD due to hypertension is an important part of reducing the total burden of CKD. In 2020, the World Kidney Day campaign emphasized the importance of preventive interventions (primary, secondary, and tertiary prevention) in preventing and delaying the development of kidney disease (41). Moreover, accurate identification of risk factors leading to CKD or faster deterioration of renal health is closely related to health policy decision making, health education, and awareness related to CKD (42).

This study has several limitations. First, more detailed information of included countries could not be obtained from the GBD database and the lack of timely updated data on explanatory variables, which may limit the comprehensive analysis of associated factors of disease burden caused by CKD due to hypertension. Second, there may be deviation in the process of stepwise multiple linear regression because there may be intra-group differences in some countries that span a large territory, such as China and India. Third, limited by the availability of data source, only the commonly used country-level indexes were selected as potential associated factors with CKD attributable to hypertension, which may limit the generalization of our findings to some extent. Nevertheless, this study can be used as a supplement to further understand the disease burden of CKD due to hypertension and as a basis for informing policies and setting priorities for action.

In conclusion, this study provides the latest and more detailed estimates of the disease burden of CKD due to hypertension from 1990 to 2019. From 1990 to 2019, the DALY numbers and the age-standardized DALYs per 100,000 population caused by CKD due to hypertension increased by 125.2 and 10.9%, respectively. Generally, being male and older age are associated with higher disease burden. Improving the achievements and equality of distribution in health, education, and income, as well as increasing the number of physicians per 10,000 people could help to reduce the burden of CKD due to hypertension. The study may be beneficial in providing relevant information for policymakers to develop improved prevention and treatment strategies for CKD due to hypertension.

## Data Availability Statement

Publicly available datasets were analyzed in this study. This data can be found here: http://ghdx.healthdata.org/gbd-results-tool, http://hdr.undp.org/en/data.

## Author Contributions

LT, GJ, and H-CC did the conceptualization, methodology, supervision, and validation. AC and MZ handled the methodology, formal analysis, data curation, writing of the original draft, reviewing, and editing of the manuscript. CY and WZ also reviewed, edited, and wrote the final manuscript. All authors contributed to the article and approved the submitted version.

## Conflict of Interest

The authors declare that the research was conducted in the absence of any commercial or financial relationships that could be construed as a potential conflict of interest.

## Abbreviations

CKD: chronic kidney disease
DALY: chronic kidney disease
HDI: human development index
HAQ: health access and quality
IHDI: inequality-adjusted human
SDI: socio-demographic index
YLD: years lived with disability
YLL: years of life lost.
